# Meteorological Table

**Published:** 1813-02

**Authors:** 


### METEOROLOGICAL TABLE.

*From December* 25, 1812, *to January* 25, 1813.

| | D. | Therm. | Barom. | Hygrom. Dry.——Damp. | Winds. | Atmos. Variation. |
|---|---|---|---|---|---|---|
| | 26 | 36 — 37 | $30^4$ — | 5 — 7 | NE. | F. C.. S. |
| | 27 | 36 37 35 | $30^4$ [illegible] | 9 — 7 | NE. | C. C.. S. |
| | 28 | 33 39 40 | $30^5$ $^4$ | 7 8 9 | W. SW. | F. C . C.. |
| | 29 | 42 46 45 | $30^3$ $^2$ | 10 — — | SW... | C.. F. C... |
| | 30 | 44 46 45 | $30^1$ — | 10 — 9 | W..SW.. | F... F... C.. |
| | 31 | 44 — 43 | $29^9$ $^7$ | 10 — 9 | SW.. | C.... R. in N |
| | 1 | 43 46 45 | $29^7$ 30 | 9 — 10 | W.. | C.... R. C... |
| ● | 2 | 43 — — | $30^1$ $^2$ | 11 — 10 | NW. | F. C.. F.... |
| | 3 | 39 40 43 | $30^2$ — | 10 — — | NW. | Fog ... F.. Fog .. |
| | 4 | 42 — 41 | $30^2$ — | 10 — 8 | SW. S. | Fog .. C... — |
| | 5 | 39 44 43 | 30 $29^9$ | 6 — 10 | SW.. | C... C.... R..in N |
| | 6 | 44 46 49 | $29^8$ $^7$ | 10 — 12 | SW... | C... R. —. |
| | 7 | 44 45 — | $29^7$ $^5$ | 14 — 10 | SW... | Fog ..C...—..R.inN |
| | 8 | 46 48 44 | $29^3$ $^5$ | 12 — 10 | SW.. | C.... F...— ... |
| ◐ | 9 | 35 39 — | $29^6$ $^7$ | 9 — 10 | NW.. | F.... C.. R... |
| | 10 | 35 38 32 | $29^8$ $^7$ | 10 — 8 | N. | F.... — ..... — .... |
| | 11 | 36 39 38 | $29^7$ — | 10 — 9 | SE.. | C... F... C... |
| | 12 | 35 — 34 | $29^8$ $^7$ | 7 — 6 | SE. | F...C.... — ... |
| | 13 | 36 40 39 | $29^6$ $^5$ | 10 — — | SE.. | C.... C.... R.. |
| | 14 | 38 — 39 | $29^6$ $^7$ | 10 — 8 | SE. | C.... Fog .. R.. |
| | 15 | 37 39 38 | $29^8$ $^9$ | 10 — 8 | NW.. | C.. C.... F. |
| ○ | 16 | 35 — 38 | 30 $^1$ | 8 — 10 | SE.. SW. | F... F.. — .. |
| | 17 | 37 38 34 | $30^1$ $^2$ | 10 — — | S. | C.... F.. C.. |
| | 18 | 32 — 33 | $30^2$ 30 | 10 — — | SE.. | Fog..C...— .Haze.. |
| | 19 | 33 — 35 | 30 $^2$ | 10 — — | SE. E.. | Snow . C.... — ... |
| | 20 | 35 36 — | $30^3$ — | 10 — 8 | E.. | C.... C.... — ... |
| | 21 | 34 35 36 | $30^3$ $^4$ | 8 — 7 | E.. | C.... — .... Snow . |
| | 22 | 34 — 30 | $30^4$ — | 8 — 6 | SE... | C.... F.. — .... |
| | 23 | 29 35 — | $30^3$ $^2$ | 8 — — | N. NE. | F... C. Snow ... |
| ◑ | 24 | 32 35 33 | $30^3$ — | 8 — — | NE.. | C.... — .. — .. |
| | 25 | 31 34 35 | $30^4$ — | 8 — 6 | NE . | F.. C.... — .... |

Quantity of rain from December 25, 1812, to January 25, 1813, $\frac{49}{100}$ of an inch.

The character of this interval has been an extraordinary degree of dryness. A period of thirty-one successive days, in the middle of winter, affording scarcely half an inch of rain, is a remarkable occurrence in this climate. From December the 26th, 1811, to January the 26th, 1812, the quantity of rain was one inch $\frac{43}{100}$, nearly treble the quantity of the present interval.

On the 19th a small quantity of snow, followed by a dense fog: scarcely light at ten o'clock a. m. On the 24th snow thinly covering the streets and houses, being the first and only fall of snow that has yet so done.

As usual at this season, pulmonic complaints are frequent. Several cases of diarrhœa have occurred, both in adults and infants. A boy about twelve years of age, who had long been subject to a violent pain in the head with occasional fits, irregular appetite, and constipation, was cured by purgatives repeated every other day for the space of a month. The fæces, which were hard, and of a black hue, regained a healthy appearance; a blister to the head much relieved the pain, but the disease was not materially amended till after the exhibition of several purgatives.

MONTHLY

